# 

**Published:** 1857-10

**Authors:** 


### NOTES AND CORRESPONDENCE.

The Editor is anxious to give free scope to the expression of opinion, but does not hold himself responsible for the opinions of those of his contributors who attach their names to the papers furnished by them.

Ague Queries. We have received from Dr. Brown of Chatham, Mr. Rigden of Canterbury, and Dr. Grantham of Burgh-le-Marsh, elaborate reports in answer to the queries on ague published in last number: for which we return our best thanks. These papers are held in reserve, in expectation of others during the ensuing quarter. We shall then classify them, and place the labours of our collaborateurs in this department in one special report.

Condy's Disinfecting Fluid. We have received specimens of this disinfectant, with various testimonials as to its efficiency. As the value of this agent is at the present time being subjected to experiment, we are not prepared to discuss its merits on this occasion. A report on it will probably appear in next number.

The Wandsworth Sanitary District. The Medical Officers of Health for the Wandsworth district, after labouring for the sanitary improvement of their respective parishes, and producing a series of very able reports, have come under the ban of their penny-wise-and-pound-foolish Board. Their salaries are to be reduced to an absurd minimum; and even their motives are questioned by the narrow-sighted politicians whose servants they unfortunately are. We recommend these political gentlemen to learn the motto on our title-page.

The admirable Irish Census Report, furnished for review in this Journal, is not forgotten. The review is simply delayed in consequence of the labour required for analyzing so voluminous a document, and extracting from it the valuable inferences which it supplies.

Cholera. We are informed by our correspondent Dr. Webster, who has just returned from an extensive tour in Scandinavia, that epidemic cholera prevails with much virulence throughout the northern districts of Europe. At Upsala and Stockholm, especially at the former city, the malady has proved unusually severe and fatal. At Christiansand in Norway, in Copenhagen, and at Corsœr, on the west coast of Zealand, it has also proved very violent. We trust these outbreaks are not the forerunners of the disease in these islands.

Preservation of Food. Dr. Spence, of Lerwick, having seen in the January number of this Review, sixteen years mentioned as a long period with regard to the preservation of animal food, sends us a note of the following instance, in which animal food was perfectly wholesome after more than twice that length of time.

"In October 1823," says Dr. Spence, "when Parry returned from his second Arctic Expedition to Lerwick Harbour, he presented to my father, the late William Spence, Esq., Surgeon to the Forces, two tins of preserved soup, with a request that they might be kept unopened as long as the contents could be reasonably expected to continue sound and fit for use. They were put aside and almost forgotten, until the 14th of July of the current year; when, having read the article on the Preservation of Food in No. VIII of the Sanitary Review, I was reminded of their existence, and determined on opening them. The insides of the tins were white and untarnished, and the contents were free from any unpleasant odour or flavour. One of them contained concentrated meat soup, and the other vegetable soup. I submitted them to various individuals, who though at first rather chary of tasting them, declared them to be perfectly fresh. I had the contents of the two tins mixed and warmed for dinner, and I certainly never tasted better soup.

"The expedition sailed from England on the 29th of April, 1821, so that the soups must have been rather more than thirty-six years old, and must have passed two winters in the Arctic regions.

"In the published account of the expedition, it appears that these soups were prepared by Messrs. Gamble & Co.

"Lerwick, 7th September, 1857."

The Tucker Fund is progressing favourably; and a subscription list will soon be printed. We owe thanks to those gentlemen who have benevolently responded to our last appeal.

The papers of Dr. Pickells and Mr. Cox are received, but are unavoidably delayed.

Dr. Richardson would be obliged if correspondents would kindly address their papers and books for review to his private residence, 12, Hinde Street, Manchester Square, London, W.

### BOOKS RECEIVED.

Acton (William). A Proposal to form a London Female Sanitary Society and Savings Bank. London: 1857.

Aldis (C. J. B., M.D., M.A.) A Letter to the Vestry of St. George's, Hanover Square, on Sanitary Work and Experience in Belgravia. London: Rice, 1857.

Aldis (C. J. B., M.D.) Lecture on the Sanitary Condition of Large Towns, and of Belgravia. London: Rice, 1857.

Bertillon (Le Dr.) Conclusions Statistiques contre les Detracteurs de la Vaccine. Paris: Victor Masson, 1857.

Board of Works for the Wandsworth District. First Annual Report on the Sanitary Condition of the several Parishes comprised in the Wandsworth District during the Year 1856. London: 1857.

Cosmos: Revue Encyclopédique Hebdomadaire des Progrès des Sciences. July 31st, 1857.

Evans (Conway, M.B.) First Annual Report of the Sanitary Condition of the Strand District. London: 1856-7.

Gamgee (J. S.) Two Letters to Lord Palmerston on Medical Reform, a Social Question. London: Blackie and Sons.

Gibbon (Septimus, M.D.) Reports to the Board of Works for the Holborn District from Feb. 1856, to March 1857. London: 1857.

Glaisher (James, F.R.S.) On the Meteorology of England for the Quarter ending June 30, 1857. London: 1857.

Godrich (Francis). Report on the Sanitary Condition of St. Mary Abbot's, Kensington. Kensington: 1857.

Lindsay (Lauder, M.D.) Suggestions for Observations on the Influence of Cholera and other Epidemic Poisons on the Lower Animals. Edinburgh: 1857.

Lobb (H. W.) On the Use of Mincasea, a Prepared Milk for Infants. London: 1857.

Mackay (George, M.D.) Notes on the Cholera at Varna in 1854, and more especially in Her Majesty's Ship *Agamemnon*, in the Black Sea. Edinburgh: Murray and Gibb, 1857.

Marcet (W., M.D., F.C.S.) On the Fatty Matters of Human Excrements in Disease. London: 1857.

Nicholas (G. E.) Report on the Sanitary Condition of Wandsworth for the Year 1856.

Northcote (A. B., F.C.S.) On the Constitution of Allophane. London: 1857.

Oesterlen (F., M.D.) Handbuch der Hygiene. Tübingen: 1857.

Poznanski (F. X., M.D.) De la Nature, du Traitement, et des Préservatifs du Cholera. Deuxième Edition. Paris: Baillière, 1857.

Pearce (Samuel). Report on the Sanitary Condition of St. Matthew, Bethnal Green. London: 1857.

Questions of the Day. By the Creature of an Hour. London: Longmans, 1857.

Report (Thirteenth Annual) of the Directors of Murray's Royal Asylum, Perth. Perth: 1857.

Ross (George). City Dwellings for the Working Classes. London: 1857.

SALTER (HYDE, M.D.) An Introductory Address delivered at King's College, London, October 1857. London: 1857.

SIMON (John, F.R.S.) Papers relating to the History and Practice of Vaccination. London: Blue Book, 1857.

Speech on Medical Reform, delivered by Lord Elcho, M.P., in the House of Commons, July 1, 1857. London: T. Hatchard. 1857.

STEPHENS (Henry). Cholera: an analysis of its Epidemic, Endemic, and Contagious Character, and on the Treatment of Fevers generally, and more especially Cholera. London: Renshaw, 1849.

TABLES Referring to Indian Prisons.

WESTALL (E.) Report on the Mortality of Croydon for Quarter ending June 30, 1857.

WHITEMAN (R. H.) Report on the Sanitary Condition of Putney and Roehampton for the Year 1856.

WILSON (J. G., M.D.) Cases showing the Advantages of Turning as a Substitute for Craniotomy and the Long Forceps in Cases of Pelvic Contraction. Glasgow: 1857.

---

### IN EXCHANGE.

Literary Gazette.
Psychological Journal.
British Medical Journal.
Ranking and Radcliffe's Abstract of the Medical Sciences.
Chemist.
Veterinarian.
Asylum Journal of Mental Science.
Quarterly Journal of the Chemical Society.
Scottish Review.
Edinburgh Medical Journal.
Glasgow Medical Journal.
Dublin Hospital Gazette.
American Journal of the Medical Sciences
New York Journal of Medicine.
Montreal Medical Chronicle.
Charleston Medical Journal.
Gazette Médicale de Paris.
Aerzliches Intelligenz-Blatt.

---

### To New Subscribers to the Sanitary Review.

THE Publisher begs to intimate, that new Subscribers wishing to have the series of the Journal complete from the first number, can obtain sets of Vols. I and II, at Subscribers' prices, 10s. each, post free, by application to him, enclosing Post-office Order, payable at the Charing Cross Branch to THOMAS RICHARDS.

THE SANITARY REVIEW AND JOURNAL OF PUBLIC HEALTH is sold by all Booksellers, and is equally suited for the general and professional reader, for the scientific society, and for the private library.

London: T. RICHARDS, 37, Great Queen Street.

